# Ligand-based and structure-based design of novel histone demethylase inhibitors

**DOI:** 10.1186/1758-2946-5-S1-P41

**Published:** 2013-03-22

**Authors:** Luca Carlino, Martin Leo Schmitt, Manfred Jung, Wolfgang Sippl

**Affiliations:** 1 Institut für Pharmazeutische Chemie, Martin-Luther-Universität Halle-Wittenberg, Halle/Saale 06120, Germany; 2 Institut für Pharmazeutische Wissenschaften, Albert-Ludwigs-Universität Freiburg, Freiburg, 79104, Germany

## 

The genetic information in eukaryotic cells is organized in a specific structure called chromatin. The basic unit of chromatin is the nucleosome, which consist of four histone proteins and ~147 bp of DNA [1]. The N-terminal tails of these proteins contain sited for post-translational modifications directly linked to gene expression. The modifications include acetylation, methylation, phosphorylation, ubiquitinylation, sumoylation and ribosylation. Specific enzymes mediate each modification. LSD1 (Lysine Specific Demethylase I) is one of the histone demethylases, which removes one methyl group from mono- or di-methylated lysine residue. It has recently been demonstrated that androgen receptor (AR)-LSD1 complex demethylates a repressive histone mark (H3K9) promoting genes activation [2]. Experimental data show, also, that LSD1 is strongly expressed in prostate cancers [3]. For these reasons, specific modulation of LSD1 might be a promising therapeutic strategy in tissues where AR has a key physiological role. LSD1 is a flavin-dependent amine oxidase, which shares sequence identity with other flavin dependent amine oxidases like monoamine oxidase (MAO), and polyamine oxidase (PAO). After we analyzed structural differences and similarities among these enzymes several docking studies were evaluated using the available crystal structures of LSD1 and the related oxidases to discover novel LSD1 inhibitors. For the evaluation studies we selected different ligand data sets containing known inhibitors of MAO and PAO. The docking setup that showed the highest accuracy and enrichment factors was selected for virtual screening of LSD1 inhibitors. Preliminary biological data were obtained and will be discussed in the context of the target structure.
